# Factors influencing the patient safety climate in intensive care units: cross-sectional study

**DOI:** 10.1186/s12912-021-00643-x

**Published:** 2021-07-08

**Authors:** Ranielle de Lima Silva Nunes, Ana Elisa Bauer de Camargo Silva, Juliana Carvalho de Lima, Dayse Edwiges Carvalho, Cristina Alves Bernardes, Tanielly Paula Sousa, Fernanda Raphael Escobar Gimenes, Ana Claudia Andrade Cordeiro Pires

**Affiliations:** 1 Faculty of Nursing, Postgraduate Program in Nursing, Federal University of Goiás, Goiânia, GO Brazil; 2 Faculty of Nursing, Federal University of Goiás, Rua 227, s/n, Qd. 68, Setor Leste Universitário, CEP, Goiânia, GO 74605-080 Brazil; 3 Ribeirão Preto College of Nursing, WHO Collaborating Centre for Nursing Research Development, University of São Paulo, Ribeirão Preto, SP Brazil; 4 Institute of Tropical Pathology and Public Health, Postgraduate Program in Collective Health, Federal University of Goiás, Goiânia, GO Brazil

**Keywords:** Intensive care unit, Multidisciplinary team, Organizational culture, Patient safety, Safety management

## Abstract

**Background:**

Measuring the patient safety climate of a health service provides important information about the safety status at a given time. This study aimed to determine the factors influencing the patient safety climate in Intensive Care Units.

**Methods:**

An analytical and cross-sectional study conducted in 2017 and 2018 in two adult Intensive Care Units of a Brazilian Teaching Hospital. The Safety Attitudes Questionnaire instrument was applied with the multidisciplinary teams to determine the factors influencing the patient safety climate. Data were double entered into a database and processed using the R (version 3.5.0) statistical software. Position, central tendency and dispersion measures were taken and absolute and relative frequencies, mean and confidence intervals were calculated for the quantitative variables. Linear regression was performed to verify the effect of variables on the SAQ domains. Variables with a *p*-value of less than 0.25 were selected for multivariate analysis.

**Results:**

A total of 84 healthcare providers participated in the study. The mean Safety Attitudes Questionnaire score was 59.5, evidencing a negative climate. The following factors influenced the safety climate: time since course completion, professional category, type of employment contract, complementary professional training, and weekly workload.

**Conclusions:**

The factors identified indicate items for planning improvements in communication, teamwork, work processes, and management involvement, aiming to ensure care safety and construct a supportive safety climate.

## Background

Patient safety has been discussed worldwide and refers to the reduction of the risk of unnecessary healthcare-related harm [1]. The safety culture is the central element of many initiatives aimed at preventing care failures [2] and refers to the product of individual and group values and patterns of behaviour that determine the commitment and management style of an organization [3].

Measuring the patient safety climate is a way of assessing the culture, as it portrays the perceptions of the employees at a given time and provides important information about the safety status [4]. A positive safety climate allows for better risk management, for a reduction in the number of adverse events, and for better patient outcomes [5]. Conversely, a poor safety climate can influence the adherence of the professionals to the best practices and compromise quality of care and patient safety [5].

The Intensive Care Unit (ICU) is a favourable environment for the occurrence of adverse events due to the complexity of the care provided, the severity of the patients treated in these units, and the work performed, often under stressful conditions and with the involvement of a multidisciplinary team [6]. In a study conducted in USA, one in four critically ill patients suffered some adverse event during their stay in the ICU, with the positive patient safety climate score being related to a reduction in hospital mortality, length of stay, and the rate of healthcare-associated infections [7].

Considering the relevance of the theme, studies have been carried out in order to identify the safety climate in ICUs [8, 9]. However, in order to construct the path of cultural change, with a view to establishing a positive patient safety climate, it is necessary to go beyond identifying the climate. It is imperative to know the organizational factors that may be influencing or preventing the development of a safety culture favourable to the reduction of healthcare-related adverse events [4, 10]. The approval of the teams in relation to management actions, collaboration among the staff, job satisfaction, and the quality of the work environment and logistical support are positive factors for the patient safety climate [11]. Improving the competence of nurses for patient-centred care and creating a strong safety climate are important to promote synergy between patient participation and safe practices [12]. This way, the aim of this study was to identify the factors influencing the patient safety climate in ICUs.

## Methods

Analytical and cross-sectional study carried out using two data collection instruments:

### Healthcare provider characterization form

To characterize the healthcare providers, a questionnaire was applied. The response variables evaluated in the study were: teamwork climate; safety climate; job satisfaction; stress recognition; perceptions of the management unit and hospital; and working conditions. The explanatory variables were: age, time since course completion (technical or graduate course), time working in the current sector, time working in the institution, number of extra shifts per month, work unit, sex, professional category, complementary professional training, type of employment contract, weekly workload, and extra duty performed in the institution.

### Safety attitudes questionnaire – SAQ

The Safety Attitudes Questionnaire – SAQ [13–15] is a self-administered questionnaire for assessing the safety climate, with 41 items, divided into six domains, namely: Teamwork Climate, Safety Climate, Job Satisfaction, Stress Recognition, Perceptions of Management, and Working Conditions. Each item is answered on a five-point Likert-type scale: A = strongly disagree, B = slightly disagree, C = neutral, D = slightly agree and E = strongly agree. Items 2, 11, 20, 21, 22, 23, and 36 were reverse scored [16].

### Data collection

The study was conducted in two ICUs of a public teaching hospital located in the state of Goiás, Brazil. The Adult ICU 1 had eight beds for surgical patients. The Adult ICU 2 had six beds for clinical patients. The study population was constituted by all the healthcare providers working in the multidisciplinary teams of the ICUs, with 58.3% belonging to the Adult ICU 1. The sample was by convenience/non-probabilistic and the response rate was 94.0%.

The healthcare providers that participated in the study were: physicians, registered nurses, nurse technicians, physiotherapists, a dietician and psychologists. In Brazil, there are three categories of nurses: registered nurses, who have graduated and are responsible for the supervision of the other two categories; nursing technicians, who perform medium and high complexity procedures; and nursing assistants, who provide basic care to patients [17].

The healthcare providers included in this study were those that were working in the ICU from December 15th, 2017 to February 15th, 2018, performing activities for more than 4 months at the institution. Those that were on sick leave or were transferred from the sector during this period were excluded. The healthcare providers that did not return the completed questionnaire after three attempts to collect this data were considered losses.

In Brazil, as in other countries, there are different specific rules and conditions according to the type of employment relationship. There are two types of employment contracts in the hospital: permanent employment contract regulated by the Labor Code and permanent employment contracts regulated by the federal authority [18, 19]. Statutory employment is governed by a set of special rules, which includes, among other things, lifetime contracts from which employees cannot be discharged, except for misconduct. It should be highlighted that the Consolidation of Labour Laws (CLL) was enacted to consolidate the Brazilian labour legislation in 1943. Employees under this regime do not enjoy the same level of stability. Both, statutory employees and those regulated by the CLL are hired through an open competitive examination, for which a determined level of formal education is required [19].

The healthcare providers were approached in their workplaces and received an envelope containing: a consent form, a copy of the self-administered participant characterization questionnaire and the Safety Attitudes Questionnaire – SAQ. A folder was left in each ICU for the providers to put the completed instrument in, aiming to guarantee the anonymity of the participants. The forms inside the folders were collected daily by the researcher.

Data were double entered into a database and processed using the R (version 3.5.0) statistical software. To calculate the scores, initially, the reverse items were recoded, then the items were grouped into domains and, subsequently, the sum of the items answers in each domain was calculated and the result was divided by the number of items [13–16].

The SAQ items were evaluated on a scale from 0 to 100. Those items that obtained scores of 75 or more were considered indicative of a positive patient safety climate. In order to easily interpret the results, the Likert score was converted to a percentile score, with a Likert score of A corresponding to 0, B to 25, C to 50, D to 75 and E to 100 [15, 16]. The scores of all the items in one subscale were summed, then divided by the number of items in that subscale to obtain the score of that subscale, ranging from 0 to 100. Overall, the SAQ had good internal consistency (Cronbach’s α = .70).

In the analysis of the quantitative variables, measures of position, central tendency and dispersion were used, the boot-strap confidence interval being one of these. For the qualitative variables, absolute and relative frequencies were used. Mean and confidence interval (95%CI) were also calculated. Linear regression was used to verify the effect of the variables on the SAQ domains, with robust standard errors for the covariance matrix of the estimated coefficients.

The variables that presented a *p*-value below 0.25 in the univariate analysis, i.e. “professional category, complementary professional training, type of employment contract, weekly workload and time since course completion”, were selected for the multivariate analysis. For the Backward method, a 5% significance level was adopted. The following tests were performed: Regression coefficient, Standard error, and 95% confidence interval.

The study was conducted after approval by the Ethics Committee on Human and Animal Medical Research of the institution (number 1.887.147) and carried out following the research recommendations [20].

## Results

A total of 84 healthcare providers participated in the study; 72.6% were female, with a mean age of 43.7 years; 73.8% had an employment contract regulated by the federal authority, with a mean time of 12.8 years working in the institution and 10.3 years in the ICU. Regarding working hours, 73.5% worked 30 h per week, 7.2% 36 h, and 9.6% 40 h. The nursing care providers with an employment contract regulated by the federal authority worked a mean of 3.5 extra shifts at the units per month.

Of the study participants, 56.0% were nursing technicians, 20.2% registered nurses, 19.0% physicians, and 4.8% belonged to other categories (physiotherapists, dietician and psychologists). The mean complementary professional training was 19.22 years. The providers with master’s or doctoral degrees correspond to 20.0% and, 65.7% had completed some type of specialization course. Among the nursing technicians, 21.8% had a degree and 47.8% were specialists.

The general SAQ score identified in the present study was 59.5, indicating a negative safety climate. The Job Satisfaction domain was the only one that presented a positive score (81.8). The lowest scores were verified in the Working Conditions (43.4) and Perceptions of Management domains, as presented in Table 1.

**Table 1:** Analysis of the general safety climate in two ICUs by domain

Domains	Mean	95% CI^a^
General Score	59.5	[56.2; 62.7]
Teamwork Climate	69.2	[65.0; 73.3]
Safety Climate	58.9	[54.6; 63.3]
Job Satisfaction	81.8	[77.6; 85.7]
Stress Recognition	74.8	[69.6; 79.9]
Perceptions of Management	48.8	[43.8; 53.6]
Unit	55.4	[50.0; 60.6]
Hospital	42.0	[37.4; 47.3]
Working Conditions	43.4	[37.4; 49.0]

^a^ CI: 95% Confidence Interval

The multivariate analysis data are shown in the following tables. Table 2 presents the factors that exerted an influence on the Teamwork Climate and Safety Climate domains.

**Table 2:** Factors that exerted an influence on the Teamwork Climate and Safety Climate domains

Variables	Teamwork Climate			Safety Climate		***p***-value^**c**^
	***β***^**a**^	95% CI^**b**^	***p***-value^**c**^	***β***^**a**^	95% CI^**b**^	
Professional category						
Physician	–	–	–	–	–	–
Registered nurse	−7.52	[−18.28; 3.24]	0.176	15.85	[2.31; 29.39]	**0.024**
Nursing Technician	−5.10	[−13.35; 3.16]	0.231	10.54	[−1.38; 22.45]	0.087
Others^d^	−28.88	[−46.98; − 10.78]	**0.003**	− 4.01	[−16.72; 8.7]	0.538
Complementary professional training						
Master’s degree/PhD	14.54	[4.89; 24.19]	**0.004**	–	–	–
Type of employment contract						
Consolidation of Labour Laws (CLL)	3.82	[−5.91; 13.56]	0.445	–	–	–
Employment Contracts Regulated by the Federal Authority	−11.39	[−20.92; − 1.87]	**0.022**	–	–	–
Safety Climate	0.45	[0.26; 0.65]	**0.000**	–	–	–
Working Conditions	–	–	–	0.38	[0.21; 0.55]	**0.000**
*R*^2^	36.2%			30.1%		

^a^β: Regression coefficient; ^b^95% CI: 95% Confidence Interval; ^c^Calculated using the HC matrix; ^d^ physiotherapists, dietician and psychologists

The physiotherapists, dietician and psychologists had a worse perception (−28.88) of teamwork when compared to the physicians, demonstrating that belonging to another professional category reduced the Teamwork Climate by 28.8 points. However, the nurses had a better perception of the safety climate (+ 15.85) than the physicians (*p =* .024), demonstrating that belonging to the nursing team increased the Patient Safety Climate by 15.85 points. In addition, Master’s and doctoral degree holders had a better perception of the Teamwork climate (14.54) in relation to those individuals with graduation or specialization.

The healthcare providers with employment contracts regulated by the federal authority had a more negative perception (11.39) of the Teamwork climate in relation to those with employment contracts regulated by the CLL (*p =* .022). The providers with employment contracts regulated by the federal authority had been working in the institution for longer, had higher wages, less workload, and greater job stability when compared to the providers with employment contracts regulated by the CLL. For each point added in the Safety Climate, there was an increase of 0.45 points in the Teamwork Climate.

The professional category and the Working Conditions were able to explain 30.1% of the variability in the Safety Climate. For each point added to the Working Conditions, there was a mean increase of 0.38 points in the Safety Climate (*p =* .000).

The factors influencing the Job Satisfaction and Stress Recognition domains are presented in Table 3.

**Table 3:** Factors that exert an influence on the Job Satisfaction and Stress Recognition domains

Variables	Job Satisfaction			Stress Recognition		***p***-value^c^
	*β*^a^	95% CI^b^	***p***-value^c^	β^a^	95% CI^b^	
Weekly Workload (30 h a week)	**–**	–	–	–	–	–
Weekly Workload (from 36 to 40 h a week)	–	–	–	− 14.87	[−25.11; − 4.62]	**0.006**
Time since Course Completion	−0.62	[−1.22; − 0.03]	**0.043**	–	–	–
Working Conditions	0.30	[0.11; 0.48]	**0.002**	–	–	–
*R*^2^	17.20%			7.90%		

^a^β: Regression coefficient; ^b^95% CI: 95% Confidence Interval; ^c^Calculated using the HC matrix

With each year added to the time since training, there was a reduction of −0.62 points in Job Satisfaction. In addition, the climate related to Job Satisfaction was positively associated with Working Conditions, with each point added to this domain increasing satisfaction by 0.30 points.

The providers that worked 30 h a week (*p =* .006) had better Stress Perception compared to those that worked from 36 to 40 h. The factors influencing the Perceptions of Management and Working Conditions domains are presented in Table 4.

**Table 4:** Factors that exert an influence on the Perceptions of Management and Working Conditions domains

Variables	Perceptions of Management			Working Conditions		***p***-value^c^
	*β*^a^	95% CI^b^	***p***-value^c^	***β***^a^	95% CI^b^	
Graduation/Specialization	–	–	–	–	–	–
Master’s degree/PhD	–	–	–	−14.41	[−28.78; − 0.04]	**0.054**
Safety Climate	0.58	[0.37; 0.79]	**0.000**	–	–	–
*R*^2^	26.4%			3.7%		

^a^*β*: Regression coefficient; ^b^95% CI: 95% Confidence Interval; ^c^Calculated using the HC matrix

For each point added to the Safety Climate, there was an increase of 0.58 points in the Perceptions of Management domain (*p =* .000). Individuals with master’s or doctoral degrees had a more negative perception of the climate related to Working Conditions (−14.41) than those with a degree or specialization.

## Discussion

The study evidenced a negative safety climate in both ICUs. When unfavourable, the climate can influence the healthcare providers’ adherence to the best practices and compromise quality of care and patient safety [5]. The safety climate has also been identified as unfavourable in other studies that used the same instrument, from the perspective of the multidisciplinary team in the ICU [9, 21].

The dietitian, psychologist, and physiotherapist had worse perceptions of the Teamwork climate when compared to the physicians. Safety climate surveys have shown that physicians score higher in their safety dimensions, both in the general score, and in some domains, including the Safety Climate, Teamwork Climate and Perception of Management [22–25].

Promoting teamwork among healthcare providers, addressing the communication between members, awareness of its importance, cooperation, commitment and respect among members, in a multidisciplinary way, is necessary for a strengthened safety climate. Teamwork is complex and represents an important strategy to overcome difficulties in situations that require rapid actions by members in which there is coordination and understanding of the role of each one, ensuring the quality and safety of the care provided, especially in unexpected situations and emergencies [22, 26].

The science of patient safety emphasizes that a well-developed and cohesive multidisciplinary team provides better care outcomes, including length of stay in the units, and that it also increases patient satisfaction [27]. Accordingly, some management tools and models can facilitate this process, such as the implementation of multidisciplinary clinical protocols, therapeutic plans, clinical care goals, and multidisciplinary rounds [28].

Another finding of the study highlighted the more positive perception of the Safety Climate that the nurses had compared to the physicians. However, it appears that this data may vary. A number of studies in Australian ICUs using the SAQ revealed that the intensive care physicians perceived a better patient safety climate than the nurses [22].

To considering that there is dedication to patient safety in the organization, understanding that failures are often caused by systemic problems, as well as treating errors as opportunities for improvement, are issues that have a great influence on the patient safety climate [29]. Fear of blame, punishment and retaliation must be replaced by measures to make open dialogue about mistakes comfortable for the providers, improving their perception of the Safety Climate and contributing to the prevention of errors [30].

In this study, the nurses’ perception of a better climate could be a result of their involvement in planning patient safety actions in the organizations. Nursing has been a pioneer in the educational path toward patient safety, with efforts having been focused on demonstrating the importance of multidisciplinary involvement [21]. The nurses have invested in strategies to develop and articulate cooperation between health institutions and education with regard to patient safety.

Professionals with employment contracts regulated by the CLL, who had been working in the institution for less time, had a better perception of the Teamwork Climate domain when compared to those providers with employment contracts regulated by the federal authority. It is believed that the admission of healthcare providers from different specialties can contribute to the positive modification of this scenario and to the improvement of the Teamwork Climate. Other interventions, such as improving the processes of communication, cooperation, coordination, respect, and the work climate, are also necessary for inter-professional work to take place effectively, with a participatory, collaborative, and coordinated approach to decision-making among the team [31].

On the other hand, the professionals with master’s or doctoral degrees had a more negative perception of the climate related to Working Conditions when compared to those with graduate degrees or specializations. According to other authors, these results may indicate that a higher level of education can increase the criticality of these healthcare providers in relation to problems related to patient safety at the unit and increase the demands in view of reflections on the theme, impairing their perception of the climate in their unit [3].

This result may also be associated with the fact that these providers, represented here by physicians, nurses and a dietician, are closer to positions of diagnostic decision-making, interventions and care, and may have impairments in their work due to the lack of organization of the management processes, delays by the sectors in fulfilling requirements, and unavailability of reports and exams, among other aspects. In this context, the lack of standardization and preparation for the handoff, which consists of transmitting relevant information for the continuity of the patient’s treatment, including the current health status, recent changes, ongoing treatment, and the transfer of responsibility for the patient to another provider or team, can lead to delayed, wrong or missed procedures [32]. One way to support clinical decision-making, based on improving communication among teams, is to structure communication strategies using tools such as the SBAR-Situation-Background-Assessment-Recommendation [33]*.*

The results of this study also revealed that, more time since course completion equated to less Job Satisfaction. Institutions that present a sub-optimal level of professional satisfaction often have increases in staff turnover and the occurrence of adverse events, including falls, infections, and medication errors [34]. One study showed that older healthcare providers, mainly women, were more likely to be dissatisfied with their work. Lower Job Satisfaction can also be associated with other contexts, including extra hospital factors. Many providers have another employment contract and, when referring to working women, this is often associated with domestic chores, which can cause tiredness and exhaustion [35].

The providers with workloads greater than 30 h (36 h and 40 h) perceived less stress than those that worked 30 h per week. The hypothesis for this finding is associated with the fact that most providers that work 30 h (73.5%) have an employment contract regulated by the federal authority. These professionals can work extra shifts in the units, with a mean of 3.5 extra shifts per month found in the present study. Studies conducted in Liaoning Province, China, and the Gaza Strip found that increased weekly working hours triggered lower patient safety climate scores [3, 30]. The increase in the working hours and voluntary overtime has been related to the increased likelihood of adverse events [36]. Work overload can lead to an increase in the length of patient stay and to a greater risk of death [37].

The Safety Climate influenced the Perceptions of the Management domain, contributing to 26.34% of its variability, which makes it possible to infer that the involvement of the management is of paramount importance for the construction and dissemination of the safety culture. Organizational leaders are decisive in developing a positive climate for the professional practice [8, 38]. A management model that fulfils the needs of all involved, providers and patients, can collaborate to strengthen the patient safety climate, in addition to providing coordinated, effective, and safe work for all [39]. The planning, implementation, and evaluation of the improvement actions in order to strengthen the safety climate must be associated with feedback, also considering organizational attitudes, infrastructure, and social and contextual awareness, for the interventions to be successful [39].

The results showed that, for each point added to the Working Conditions, there was a mean increase of 0.38 points in the Safety Climate, which demonstrates that these domains are related, and that improvements related to Working Conditions will result in a better Safety Climate.

In the present study, the providers that worked fewer hours presented better Stress Perception scores compared to those that worked more. In addition, the increase in the number of hours worked per week resulted in a reduction in the employee’s perception of the negative impact of stress on their work. Therefore, the employees were less likely to perceive stress as affecting their performance and contributing to unsafe situations [40, 41].

The important influence of human factors on patient safety can be perceived, as well as the assessment of the safety climate at the local level, seeking improvement strategies that are adequate to the reality of the unit and focused on its particularities. The role of the safety climate must be emphasized at all levels of management, with a multidisciplinary approach, seeking improved communication and cooperation within teams [22, 41]. Accordingly, improvements in the patient safety climate within a system will contribute to sustained patient safety outcomes as well as employee satisfaction [29].

The limitations of the study include its performance in only two ICUs in the same hospital, the exclusion of non-care professionals, the analysis at a single point in time and the relatively small sample size. However, in relation to the strengths of this study: considering that nurses have the central role in improving patient safety, the study highlights the importance of building their competence during the nursing education to reinforce a strong safety culture in the clinical practice. The study also demonstrates the relationships among safety climate, transparent communication, teamwork, work processes, and management involvement, therefore emphasizing initiatives to improve the practice environment. Furthermore, the results show that nurse managers need to focus on how to empower nurses in their patient safety efforts to improve the workplace and the work practices, seeking a strong safety climate.

## Conclusion

In this study, the healthcare providers perceived a poor patient safety climate. In addition, the safety climate was related to the Perception of Management, Teamwork and Working Conditions domains of the SAQ, while working conditions were related to job satisfaction.

The innovation of the present study is in the identification of factors that influence the Safety Climate: professional category, type of employment contract, complementary professional training, time since course completion, and weekly workload. Knowing how each variable affects the ICU safety climate is the starting point for planning improvement actions.

This study will contribute to the knowledge about the components that influence the patient safety climate in the ICU, as well as provide information that may favour the construction of safe working environments, improvement in the care process and its interfaces, reduction of risks and promotion of safer and more reliable care. It is necessary to conduct new studies, so that other factors that influence the safety climate are known and understood regarding their repercussion in the care outcomes. Future studies aimed at establishing a relationship between the safety climate and the outcomes of the patients and of the healthcare providers are also recommended. Nurses have a central role in improving patient safety. The nurse managers should focus on how to empower nurses in their patient safety efforts to improve the workplace and the work practices.

## Data Availability

The datasets generated and/or analysed during the current study are available in the Figshare repository, https://figshare.com/s/b14f9cec05bae3e32ada. DOI: 10.6084/m9.figshare.14339327

## Abbreviations

ICU: Intensive Care Unit
SAQ: Safety Attitudes Questionnaire
CLL: Consolidation of Labor Laws
SBAR: Situation-Background-Assessment-Recommendation
